# Endoscopic Ultrasound-Guided Drainage without Fluoroscopic Guidance for Extraluminal Complicated Cysts

**DOI:** 10.1155/2016/1249064

**Published:** 2016-05-30

**Authors:** Hyeong Seok Nam, Hyung Wook Kim, Dae Hwan Kang, Cheol Woong Choi, Su Bum Park, Su Jin Kim, Dae Gon Ryu, Joon Ho Jeon

**Affiliations:** Department of Internal Medicine, Pusan National University School of Medicine and Research, Institute for Convergence of Biomedical Science and Technology, Pusan National University Yangsan Hospital, Yangsan 50612, Republic of Korea

## Abstract

*Background*. Endoscopic ultrasound- (EUS-) guided drainage is generally performed under fluoroscopic guidance. However, improvements in endoscopic and EUS techniques and experience have led to questions regarding the usefulness of fluoroscopy. This study aimed to retrospectively evaluate the safety and efficacy of EUS-guided drainage of extraluminal complicated cysts without fluoroscopic guidance.* Methods*. Patients who had undergone nonfluoroscopic EUS-guided drainage of extraluminal complicated cysts were enrolled. Drainage was performed via a transgastric, transduodenal, or transrectal approach. Single or double 7 Fr double pigtail stents were inserted.* Results*. Seventeen procedures were performed in 15 patients in peripancreatic fluid collections (*n* = 13) and pelvic abscesses (*n* = 4). The median lesion size was 7.1 cm (range: 2.8–13.0 cm), and the mean time spent per procedure was 26.2 ± 9.8 minutes (range: 16–50 minutes). Endoscopic drainage was successful in 16 of 17 (94.1%) procedures. There were no complications. All patients experienced symptomatic improvement and revealed partial to complete resolution according to follow-up computed tomography findings. Two patients developed recurrent cysts that were drained during repeat procedures, with eventual complete resolution.* Conclusion*. EUS-guided drainage without fluoroscopic guidance is a technically feasible, safe, and effective procedure for the treatment of extraluminal complicated cysts.

## 1. Introduction

Traditionally, the options for drainage of extraluminal complicated cysts, including peripancreatic fluid collections (PFCs), walled-off pancreatic necrosis (WOPN), and other abdominal and pelvic abscesses, have involved percutaneous or surgical approaches. Conventional endoscopic transmural drainage of pseudocysts was first reported in 1985 and subsequently widely performed [1, 2]; however, the utility of this procedure was limited in endoscopically nonvisible, infected, or persistent lesions and in patients with portal hypertension [3]. Since the introduction of endoscopic ultrasound (EUS) in the 1990s, abdominal organs in the nearby gastrointestinal tract have been easily accessed for drainage. This procedure enables access to nonbulging lesions or abscesses without luminal compression and can be performed in patients with venous collaterals and those with a small anatomic window for drainage [4, 5]. EUS is advantageous because drainage can be performed in real time under sonographic guidance [4].

Many studies have investigated EUS-guided therapy of extraluminal complicated cysts, especially pseudocysts and WOPN, and this minimally invasive technique is now regarded as a feasible option for definitive endoscopic treatment [6]. Although only a few reports have described EUS-based approaches to the drainage of other abdominopelvic abscesses, the procedure has been described as safe and effective in abscesses not amenable to drainage via various routes under ultrasound (US) or computed tomography (CT) guidance [7]. Notably, most previous studies used fluoroscopic guidance to complete drainage, and EUS-guided transmural drainage is generally performed under fluoroscopic guidance. However, a few studies have reported the results of EUS-guided drainage without fluoroscopy [8–11]. Fluoroscopic observation is mainly practical and helpful for estimating a fistula or abscess cavity and confirming proper guidewire coiling in cysts. However, X-ray assistance may expose patients and endoscopists to radiation. Additionally, in many centers, fluoroscopy and EUS examinations are performed in separate rooms, which might prevent continuative procedures. With improvements in endoscopic techniques and experience, questions have been raised regarding the necessity and usefulness of fluoroscopy, as elaborate endoscopic and EUS manipulation seem to provide sufficient coverage. In this study, we aimed to retrospectively evaluate the safety and efficacy of EUS-guided drainage without fluoroscopic control for extraluminal complicated cysts such as PFCs as well as pelvic abscess.

## 2. Patients and Methods

Between November 2012 and October 2015, 15 consecutive patients with extraluminal complicated cysts, including symptomatic large pseudocysts or WOPN, peripancreatic abscesses, and pelvic abscesses, were treated endoscopically at Pusan National University Yangsan Hospital. Four cases involved pelvic abscesses not amenable to drainage under US or CT guidance that had been referred from the surgical department because of a lack of an adequate and safe window. All patients included in the study underwent EUS-guided intramural drainage without fluoroscopic guidance following a dedicated CT scan of the abdomen and pelvis to ascertain the underlying nature and confirm the lesion. In a bleeding risk assessment, none of the patients were found to have coagulation problems (prothrombin >1.5 international normalized ratio [INR] or platelet count <50,000/*μ*L). All patients received a single intravenous dose of prophylactic antibiotics or were already receiving therapeutic antibiotics at the time of the procedure. The bowel was prepared using polyethylene glycol in the 3 patients with pelvic abscesses. Seventeen procedures were performed in the 15 patients via a transgastric (*n* = 11), transduodenal (*n* = 2), or transrectal (*n* = 4) approach. Procedures were performed under conscious sedation with intravenous midazolam and pethidine while in the left lateral decubitus or supine position. All procedures were performed using a therapeutic linear array echoendoscope (GF-UCT240, Olympus Corp., Tokyo, Japan) with working channels of 3.7 mm.

**Figure 1:**
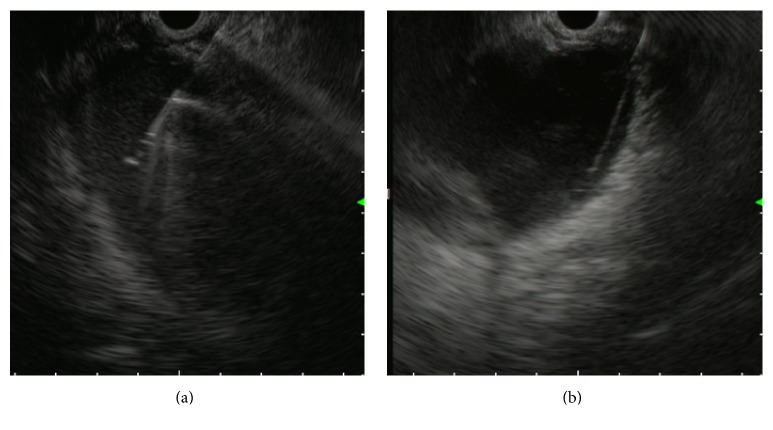
Endoscopic ultrasound (EUS) images. (a) EUS-guided puncture of a complicated cyst with a 19-gauge fine needle, and (b) placement of a 0.035-inch guidewire into the cavity.

**Figure 2:**
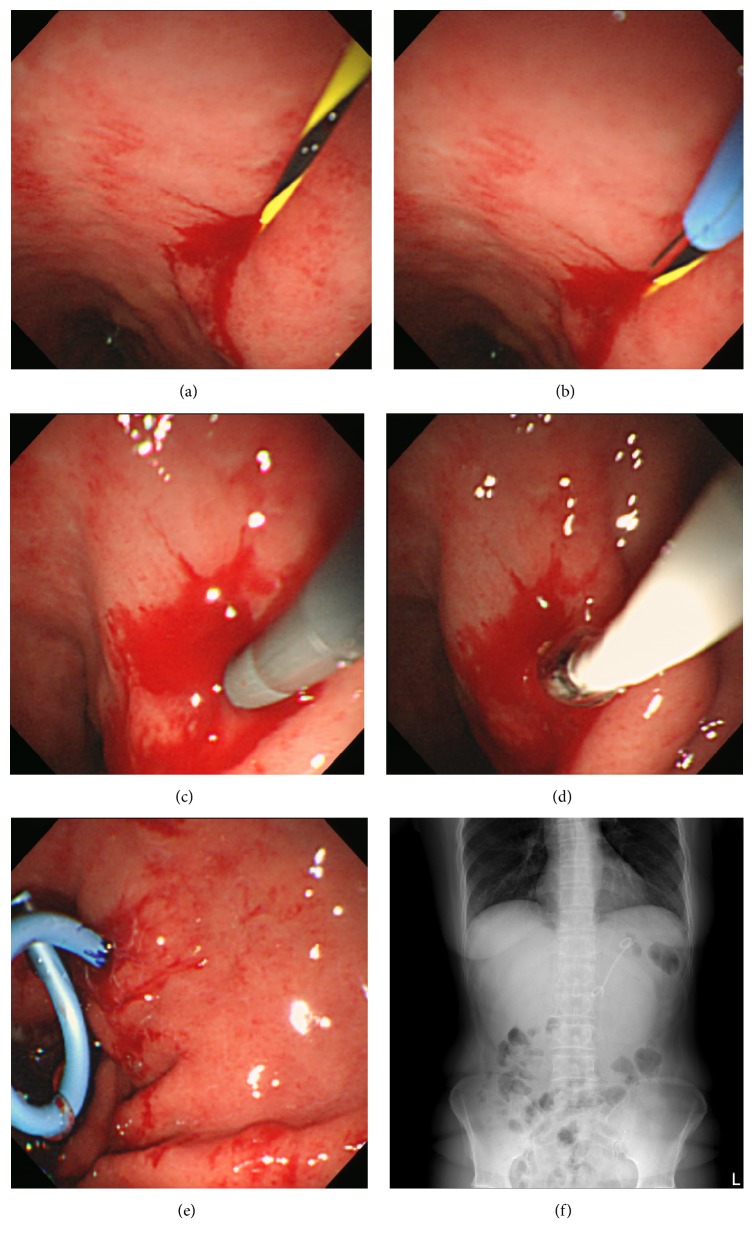
Endoscopic images (a–e) and radiologic image (f). (a) Placement of a 0.035-inch guidewire into the cavity. (b) Transmural incision using electrocautery administered via an over-the-wire needle-knife catheter under endoscopic view. (c) Bougienage with 7 Fr biliary dilatation catheters. (d) Sequential dilatation using a 4 mm × 40 mm wire-guided Hurricane RX Balloon Dilator under endoscopic guidance. (e) Transgastric placement of a 7 Fr pigtail stent to drain the pseudocyst. (f) X-ray view of a 7 Fr pigtail stent after endoscopic ultrasound-guided drainage.

EUS-guided transmural drainage of extraluminal complicated cysts was performed according to the following steps:

1. Complicated cysts, the gastrointestinal wall, and adjacent structures were subjected to EUS and endoscopic inspection. The lesion and contact area between the gut wall and cyst or abscess were located by EUS, and color flow Doppler was used to localize any regional vasculature. The minimal distance and optimal site for drainage were then identified.
2. EUS fine-needle aspiration (FNA) to puncture the cavity was performed with a 19-gauge needle (Figure 1(a)). The stylet was removed, and the contents were aspirated and sent for bacterial culture. Where possible, for cases of abscess, normal saline was flushed into the cavity to evacuate as much pus as possible.
3. A 0.035-inch guidewire was advanced through the needle until adequate resistance against the collection was achieved and was then coiled into the cavity under EUS guidance (Figure 1(b)). The needle was removed and the guidewire was left (Figure 2(a)).
4. If necessary, the transmural tract was dilated using electrocautery administered via an over-the-wire needle-knife catheter under endoscopic view (Figure 2(b)). The opening of the cystogastrostomy, cystoduodenostomy, or cystorectostomy was further enlarged via bougienage with a 7 Fr biliary dilatation catheter (Cook Medical, Bloomington, IN, USA; Figure 2(c)).
5. The transmural tract was sequentially dilated using a 4 mm × 40 mm wire-guided Hurricane RX Balloon Dilator (Boston Scientific, Marlborough, MA, USA) under endoscopic guidance (Figure 2(d)).
6. After dilatation, single or double 7 Fr double pigtail stents between 5 and 7 cm in length were placed over the wire across the fistula tract under endoscopic view (Figure 2(e)).

**Figure 3:**
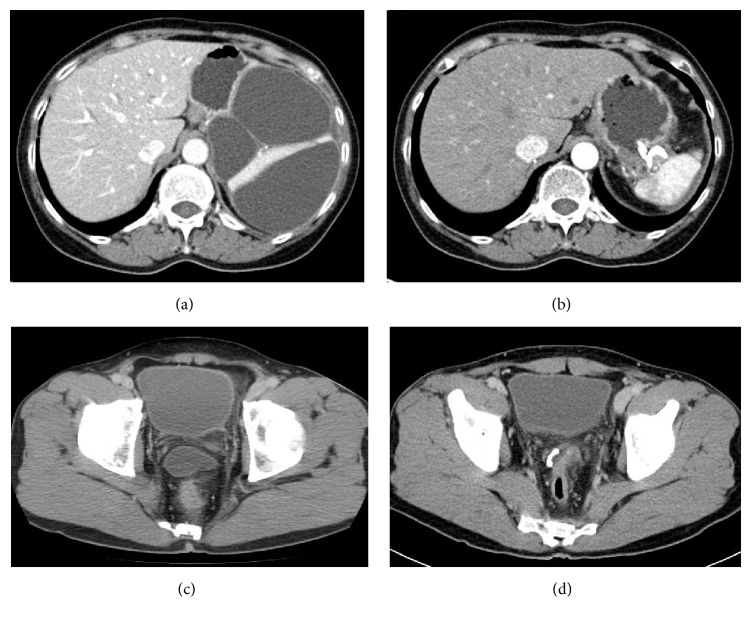
Computed tomography images (a–d). (a) A 13 cm pseudocyst in a patient with IgG4-related pancreatitis, and (b) resolution image of the pseudocyst after stent placement. (c) A 5.5 cm perirectal abscess in a patient with previous perforated diverticulitis, and (d) reduction in abscess size after stent placement.

The procedure time was defined as the elapsed time from the first image of the lesion for the EUS procedure which was obtained to the confirmed image of placement of the pigtail stent into the cyst (Figure 2(f)). Procedural success was defined as successful and appropriate placement of 1 or 2 stents in the transmural tract. Follow-up examinations, including CT, were performed within 1 month after stent placement to assess complete resolution or a decrease in the sizes of complicated cysts with clinical symptomatic improvement (Figures 3(a)–3(d)). Treatment success (or clinical success) was defined as a partial (reduction of >50% of the large axis) to complete resolution of the drained cysts with symptomatic improvement on follow-up CT at 4 weeks.

## 3. Results

EUS-guided drainage for extraluminal complicated cysts was performed in 15 patients (11 men and 5 women) with a mean age of 40.8 ± 18.0 years (range: 14–76 years) (Figure 3). Two patients had undergone the procedure twice in their lifetime for a symptomatic large pancreatic pseudocyst and a pelvic abscess. The extraluminal complicated cysts included symptomatic large pseudocysts or WOPN on the pancreas (*n* = 11), peripancreatic abscess after pancreaticoduodenectomy (*n* = 2), perirectal abscess from perforated diverticulitis (*n* = 3), and postappendectomy complication (*n* = 1). The median length of the major cystic axis was 71 mm (range: 28–130 mm). The clinical presentation and outcome of each patient who underwent EUS-guided drainage are shown in Table 1. No patient underwent percutaneous or surgical drainage before EUS-guided drainage was performed.

All procedures were performed under conscious sedation using intravenous midazolam and pethidine without fluoroscopic monitoring. The routes of approach for the procedure were transgastric (*n* = 11), transduodenal (*n* = 1), and transrectal (*n* = 3). In the case of a transgastric approach, the procedure site was the upper body of the stomach (8, posterior wall; 3, great curvature). The mean time spent per procedure was 26.2 ± 9.8 minutes (range: 16–50 minutes). Lesions were located within 1 cm of the EUS transducer, and access could be achieved in a single attempt. Stent placement was technically successful in 16 of the 17 (94.1%) procedures; in 1 patient with a peripancreatic abscess after PPPD, aspiration only was possible due to poor cooperation and the small size of the cyst (2.8 cm).

Single or double 7 Fr pigtail stents were inserted for complete drainage. In 11 patients, a single 7 Fr × 5 cm pigtail stent was deployed, and in 3 patients, a single 7 Fr × 7 cm stent was deployed. In 2 patients, double 7 Fr × 5 cm pigtail stents were deployed; in 1 of these patients with WOPN, additional percutaneous catheter drainage at another site was required because of multiple affected locations and septated pseudocysts. There were no adverse events or complications, and none of the patients required surgical intervention.

Fluid aspirate microbiological cultures from 7 of 13 pancreatic lesions showed mono- or multibacterial growth of Gram-negative (*Escherichia coli* and* Klebsiella pneumonia*) and/or Gram-positive bacteria (*Enterococcus faecium*). In 4 perirectal abscesses, both* Escherichia coli* and* Enterococcus faecium* were grown in culture from fluid aspirates. Patients continued antibiotic therapy or received adjusted therapy if the culture results indicated an infected cyst.

All patients experienced symptomatic improvement after endoscopic drainage. Follow-up CT in all patients revealed partial (>50%) to complete resolution of the drained cysts within 1 month. In 1 patient with a perirectal abscess, the stent spontaneously fell out 1 week after the procedure. Follow-up CT performed on the day that the stent fell out revealed that the abscess had decreased by >50%. The patient continued antibiotic therapy with no further drainage, and complete resolution was confirmed by CT 1 month later. The median follow-up interval was 9 months (range: 3–28 months). Two patients who had achieved an initial complete resolution of pseudocysts after endoscopic drainage developed recurrences after 1 and 3 months. These cysts were drained through repeat EUS-guided drainage procedures, and both patients eventually achieved complete resolution.

## 4. Discussion

Since the introduction of EUS in the 1990s, EUS-guided drainage has become the first treatment option for lesions such as PFCs and deep abdominopelvic abscesses. In previous studies of EUS-guided transmural drainage for PFCs or abdominopelvic abscesses, procedures were generally performed under fluoroscopic guidance [12–27]. However, the fluoroscopic view does not seem to be particularly essential in an actual practice setting, as experienced endoscopists can perform exact and proper needle puncturing under endoscopic and EUS guidance without X-ray assistance. Careful EUS inspection is also sufficient to estimate the fistula tract or the abscess cavity and visualize guidewire coiling in the cavity. After guidewire coiling, most steps are usually performed under endoscopic view. In the present study, we reported 17 cases of EUS-guided drainage for extraluminal complicated cysts without fluoroscopic control. The results were encouraging. All but 1 patient (who underwent aspiration only) experienced successful drainage, and no adverse events or complications associated with the procedure were noted.

A few previous studies reported the results of EUS-guided drainage without fluoroscopy [8–11]. In 2013, Rana et al. reported the results of nonfluoroscopic EUS-guided drainage in 20 patients with symptomatic nonbulging WOPNs [8]. The sizes of these WOPNs ranged from 5 to 16 cm. All patients experienced marked symptomatic and radiological resolution, and only 1 patient with multiple WOPNs required endoscopic necrosectomy. There were no complications associated with the procedure and no recurrences. Seicean et al. described the EUS-guided drainage of 24 patients with PFC [9]. 83.3% (20/24) drainage success rate and complete resolution were reported, and no recurrences occurred during a mean follow-up period of 18 months. Seicean and colleagues found that drainage failure was associated with a lesion diameter of <6 cm and wall thickness of >2 mm and was considered to be due to sliding of the cystotome on the pseudocyst wall. Failure was never attributed to the loss of the intracystic guidewire during stent placement. In contrast, in our study, 8 of 17 cases involved lesions <6 cm in diameter, 7 of which (87.5%) were treated successfully with stent deployment. The patient in whom drainage failed had a lesion <3 cm in diameter. Based on our data, we consider complicated cysts or abscesses >4 cm in size and lesions within 1 cm of the EUS transducer to be indications for drainage without fluoroscopic control.

In the case of pelvic abscesses, Puri et al. [10] and Hadithi and Bruno[11] demonstrated the safety and success of EUS-guided drainage of pericolic abscesses without fluoroscopic monitoring. Hadithi and Bruno [11] demonstrated that EUS-guided placement of 1 or more 7 Fr pigtail stents for pelvic abscess drainage could be safely performed without fluoroscopic monitoring and yielded excellent clinical outcomes in all 8 patients (100%). Although a single 7 Fr pigtail stent seemed to be sufficient in the majority of patients (6/8) in their series, the researchers also emphasized that the placement of a second stent without fluoroscopic guidance could be unwieldy, thus requiring further attention, and that balloon dilatation of the track would facilitate endoscopic visualization of a second stent deployment.

We compared the outcomes of 5 studies of nonfluoroscopic EUS-guided drainage, including the present study (Table 2). Considering the technique, puncture of the extraluminal complicated cyst and subsequent coiling of the guidewire into the cavity could be well visualized under EUS guidance without fluoroscopic control [8]. Compared to the fluoroscopy-guided drainage techniques, the disadvantages of EUS-guided drainage without fluoroscopy include the possibility of perforation in cases with small lesions and the risk of losing the intracystic guidewire in cases involving particularly unskilled assistants. Guidewire-induced perforation is a highly undesirable adverse event. We were able to predict the guidewire coiling state by measuring the length of the inserted guidewire and could reduce the risk of perforation with increased care throughout the procedure. According to Rana et al. [8], the guidewire was inserted slowly and no more than 10 cm of the guidewire was inserted further. Careful adherence to this method ensured that Rana and colleagues did not encounter any guidewire-induced perforation. We also aspirated cystic fluid as long as possible before stent deployment to avoid a sudden expulsion of cyst fluid after puncture or dilation of the cyst wall. By placing an appropriate amount of the air in the lumen, it is possible to ensure the field of view and thus avoid losing the intracystic guidewire. We overcame the risk of guidewire loss and correctly deployed stents using balloon dilatation of the access tract, which allowed effective endoscopic visualization, and did not experience any complications related to the procedure. Previous studies involving fluoroscopy reported technical success rates of 91–100% and complication rates of 0–52% [8–11, 24, 28]. Despite the lack of directly comparable data, some previous studies performed drainage without fluoroscopy, and our results did not differ significantly from the rates reported in those studies [8–11]. Our study included a small number of patients, single or double 7 Fr double pigtail stent placement was technically successful in 16 of 17 (94.1%) procedures (1 patient underwent aspiration alone), and no complications were reported. We found that EUS-guided drainage without fluoroscopy could be successfully performed for lesions with a diameter of >4 cm and location within 1 cm of the EUS transducer.

Our study had a few limitations. First, this was a single-center, retrospective, and noncomparative study. In addition, the sample size was small. Second, there was a possibility of selection bias. Although we included 15 consecutive patients with extraluminal complicated cysts, we might have selected cases that could have been drained without fluoroscopy. Third, regarding pelvic abscesses, all abscesses were located adjacent to the rectum. Therefore, the safety and efficacy of the technique at other colonic sites are unclear.

In conclusion, we have demonstrated the safety and efficacy of nonfluoroscopic EUS-guided drainage for extraluminal complicated cysts through our experiences and a review of the literature. Fluoroscopic guidance is helpful but does not seem to significantly influence clinical success in selected patients; therefore, the need for fluoroscopy can be obviated in some cases, allowing patients to avoid radiation exposure. Additionally, the procedure time could be minimized in centers with separate examination rooms because patient transfer to the fluoroscopic room would not be required. For more successful drainage and fewer complications, further research into optimum case selection or new techniques and stent designs will be needed.

## Figures and Tables

**Table 1:** Clinical features and outcomes of 17 procedures in patients undergoing endoscopic ultrasound-guided drainage without fluoroscopic guidance.

Sex	Age	Diagnosis	Procedure site	Location of lesion	Size of lesion (mm)	Procedure time (min)	Outcome of procedure	Number of inserted stents
M	44	WOPN	Duodenum bulb	Pancreas head	40 × 16	20	Success	1
F	54	Peripancreatic abscess	Duodenum bulb	Pancreas head	42 × 30	24	Success	1
M	76	Pseudocyst^*∗*^	Stomach UB/LC	Pancreas genu/body	50 × 50	20	Success	1
F	37	Pseudocyst^*∗*^	Stomach UB/GC	Pancreas genu/body	64 × 40	34	Success	1
M	47	Peripancreatic abscess	Stomach UB/PW	Pancreas body	28 × 28	27	Aspiration only	0
M	43	WOPN	Stomach UB/PW	Pancreas body/tail	79 × 64	50	Success	2
F	19	WOPN	Stomach UB/GC	Pancreas body/tail	101 × 65	43	Success	1
M	14	WOPN	Stomach UB/PW	Pancreas body/tail	80 × 57	16	Success	1
M	14	Pseudocyst^*∗*^	Stomach UB/PW	Pancreas body/tail	73 × 71	19	Success	1
F	61	Pseudocyst^*∗*^	Stomach UB/PW	Pancreas body/tail	70 × 65	19	Success	1
M	14	Pseudocyst^*∗*^	Stomach UB/PW	Pancreas body/tail	122 × 113	25	Success	1
F	58	Pseudocyst^*∗*^	Stomach UB/PW	Pancreas body/tail	130 × 95	32	Success	2
M	54	Pseudocyst^*∗*^	Stomach UB/PW	Pancreas tail	91 × 73	16	Success	1
M	52	Pelvic abscess	Rectum	Rectosigmoid	51 × 32	34	Success	1
M	35	Pelvic abscess	Rectum	Rectovesical fossa	55 × 35	35	Success	1
M	35	Pelvic abscess	Rectum	Rectovesical fossa	43 × 35	20	Success	1
M	36	Pelvic abscess	Rectum	Rectovesical fossa	51 × 46	19	Success	1

^*∗*^Symptomatic pseudocyst; WOPN: walled-off pancreatic necrosis; UB: upper body; LC: lesser curvature; GC: great curvature; PW: posterior wall.

**Table 2:** Comparison of outcomes among five studies of endoscopic ultrasound-guided drainage of extraluminal complicated cysts without fluoroscopic guidance.

	Our study	Rana et al., 2013 [8]	Seicean et al., 2011 [9]	Hadithi and Bruno, 2014 [11]	Puri et al., 2010 [10]
Type of study	Retrospective	Retrospective	Prospective	Retrospective	Retrospective
Number of cases (male)	17^*∗*^ (11)	20 (16)	24 (17)	8 (6)	14 (11)
Mean age in years (range)	40.8 ± 18.0 (14–76)	35.4 ± 8.4 (21–52)	53 ± 13 (17–71)	55.5 (21–74)	42 (32–55)
Type of complicated cysts	PFC (*n* = 13), pelvic abscess (*n* = 4)	WOPN	PFC	Pelvic abscess	Pelvic abscess
Median size of lesion, mm	64 × 46	100	71.5 × 28	73 × 43	73 × 66
Size < 6 cm in diameter (%)	8/17 (47)	2/20 (10)	7/24 (29.2)	2/8 (25)	14/17 (17.6)
Diameter of inserted stent (Fr)	7	7	7 or 8.5	7	10
Number of inserted stents (cases)	1 (14) 2 (2)	2 (18) 3 (2)	1 or 2	1 (6) 2 (2)	1 (9)
Technical success (%)	94.1	100	83.3	100	100^†^
Clinical success (%)	100	95	79.1	100	100
Complications (%)	0	0	16.7	0	0
Recurrence (%)	11.7	0	0	0	7.1
Median follow-up period, months (range)	9 (3–28)	14 (6–22)	18 (2–30)	38 (12–52)	6

^*∗*^17 procedures were performed in 15 patients; ^†^5 patients were cured by aspiration only with or without repeated saline flushing and therefore, a stent was not placed; PFC: peripancreatic fluid collection; WOPN: walled-off pancreatic necrosis.
